# Microvascular dysfunction in the course of metabolic syndrome induced by high-fat diet

**DOI:** 10.1186/1475-2840-13-31

**Published:** 2014-02-03

**Authors:** Cristiane Aoqui, Stefan Chmielewski, Elias Scherer, Ruth Eißler, Daniel Sollinger, Irina Heid, Rickmer Braren, Christoph Schmaderer, Remco TA Megens, Christian Weber, Uwe Heemann, Matthias Tschöp, Marcus Baumann

**Affiliations:** 1 Department of Nephrology, Klinikum rechts der Isar der Technischen Universität München, Ismaninger St. 22, Munich 81675, Germany; 2 Department of Human Molecular Genetics, Laboratory of High-throughput Technologies, Institute of Molecular Biology and Biotechnology, Faculty of Biology, Adam Mickiewicz University, Poznan, Poland; 3 Dept of Otorhinolaryngology, Klinikum rechts der Isar der Technischen Universität München, Munich, Germany; 4 Institute of Radiology, Klinikum rechts der Isar der Technischen Universität München, Munich, Germany; 5 Institute for Cardiovascular Prevention, Ludwig-Maximilian-Universität, Munich, Germany; 6 Cardiovascular Research Institute Maastricht (CARIM), Maastricht University, Maastricht, the Netherlands; 7 German Centre for Cardiovascular Research (DZHK), partner site Munich Heart Alliance, Munich, Germany; 8 Institute for Diabetes and Obesity, Helmholtz Zentrum München, Munich, Germany

**Keywords:** High-fat diet, Metabolic syndrome, Hypertension, Microvascular dysfunction, Vasoconstriction

## Abstract

**Background:**

Metabolic syndrome (MetS) is associated with increased risk of cardiovascular disease (CVD). One important feature underlying the pathophysiology of many types of CVD is microvascular dysfunction. Although components of MetS are themselves CVD risk factors, the risk is increased when the syndrome is considered as one entity. We aimed to characterize microvascular function and some of its influencing factors in the course of MetS development.

**Methods:**

Development of MetS in C57BL/6 mice on a high-fat diet (HFD, 51% of energy from fat) was studied. The initial phase of MetS (I-MetS) was defined as the first 2 weeks of HFD feeding, with the fully developed phase occurring after 8 weeks of HFD. We characterized these phases by assessing changes in adiposity, blood pressure, and microvascular function. All data are presented as mean ± standard error (SEM). Differences between cumulative dose–response curves of myograph experiments were calculated using non-linear regression analysis. In other experiments, comparisons between two groups were made with Student’s *t*-test. Comparisons between more than two groups were made using one-way ANOVA with Tukey post-hoc test. A probability value <0.05 was considered statistically significant.

**Results:**

I-MetS mice presented with weight gain, blood pressure elevation, and microvascular dysfunction characterized by augmented vasoconstriction. This finding, contrary to those in mice with fully developed MetS, was not associated with endothelial dysfunction, insulin resistance, or systemic inflammation. In the initial phase, perivascular adipose tissue showed no sign of inflammation and had no influence on the pattern of vasoconstriction. These findings suggest that the onset of hypertension in MetS is strongly influenced by vascular smooth muscle cell dysfunction and independent of important factors known to influence microvascular function and consequently blood pressure levels.

**Conclusion:**

We identified in I-MetS the occurrence of isolated augmented vasoconstriction along with blood pressure elevation, but not the presence of classical MetS components known to influence microvascular function. These findings increase our understanding of the pathophysiology of CVD risk associated with MetS.

## Background

Cardiovascular disease (CVD) is the leading cause of death worldwide. Although highly influenced by non-modifiable risk factors such as age, family history, and gender, CVD is largely caused by risk factors that can be treated or at least controlled [1]. Most of the modifiable risk factors are disorders typically associated with obesity that commonly cluster together, such as abdominal obesity, insulin resistance, dyslipidemia, and hypertension. These disorders are components of the so-called metabolic syndrome (MetS) [2]. Different clinical criteria have been proposed as being diagnostic of MetS. The National Cholesterol Education Program Adult Treatment Panel III definition is one of those most widely used [3], and defines MetS as the occurrence of three or more of the following five criteria: waist circumference over 102 cm (men) or 88 cm (women), blood pressure ≥ 130/85 mmHg, fasting triglyceride level ≥ 150 mg/dl, fasting high-density lipoprotein cholesterol level less than 40 mg/dl (men) or 50 mg/dl (women), and fasting glucose level ≥ 100 mg/dl. The association between MetS and a high risk of developing CVD is extensively reported in the literature [4-6].

Regarding the underlying processes of CVD development, key components appear to be structural and functional alterations of the microvasculature, laying the foundations for target organ damage [7]. The microvasculature consists of the smallest arteries, the arterioles, capillaries, and venules [8], therefore including the so-called resistance arteries, which consist mainly of arteries <400 μm and arterioles <100 μm in lumen diameter [9]. Dysfunction of the microvasculature has been associated with features of MetS and has been reported in different ways [10-12]. MetS components, such as insulin resistance, dyslipidemia, arterial hypertension, and abdominal obesity, can independently influence microvascular function [13-16]. In obesity, pathological adipose tissue is associated with microvascular dysfunction [16,17], and, more specifically, perivascular adipose tissue (PVAT) is now accepted to be an important modulator of vascular function [16,18,19]. Vascular anticontractile properties of PVAT were described to be mediated through the hormone adiponectin [16]. In Zucker lean rats, adiponectin provoked vasodilation of mesenteric arteries, dependent on nitric oxide (NO). In their diabetic littermates, the Zucker Diabetic Fatty rats, the vasodilation caused by adiponectin was diminished and associated with endothelial dysfunction [20]. Controversy surrounds the theory that restoring adiponectin levels is associated with improvement in endothelial function [21,22]. More recently, contractile properties of adiponectin were also identified, related to alterations in the PVAT proteome in obesity [23]. Thus, the precise characteristics of microvascular dysfunction observed in MetS remain unclear owing to the simultaneous occurrence of different disorders and their complex interaction.

In rodents, hormonal, metabolic, and cardiovascular disorders induced by hypercaloric diets are widely reported. Oliveira et al. reported that hyperglycemia, systolic blood pressure disorders, and cardiac remodeling were induced by a hypercaloric diet for 20 weeks in Wistar-Kyoto rats [24]. Sarkozy et al. reported features of MetS in Zucker Diabetic Fatty rats associated with altered cardiac expression of genes related to metabolism, signal transduction, stress response, and receptors [25]. In this study, we used the C57BL/6 mouse, which is a model widely used to study diet-induced obesity as it develops features observed in human MetS [26]. We assessed microvascular function through the mesenteric arterial arcade [27], which, in rodents, contributes substantially to regulation of peripheral resistance [28] and blood pressure [29].

We aimed to characterize features of microvascular function during MetS development in a mouse model, particularly during the initial phase when confounding factors known to influence microvascular function are not completely established. In addition, we investigated the influence of endothelium and PVAT, two important mediators of an unfavorable relationship between MetS and microvascular function.

## Methods

### Animals and diets

Male wild-type C57BL/6 J (WT) mice were housed in temperature-controlled cages (20°C to 22°C) and maintained on a 12/12-hour light/dark cycle. At the age of 12 weeks mice were randomly assigned to either a control diet (9% of calories from fat, 33% from protein, and 58% from carbohydrates) or to a high-fat diet (HFD, 51% from fat, 23% from protein, and 26% from carbohydrates). Both diets were purchased from Ssniff (Soest, Germany). We defined the group who received HFD for 8 weeks as the metabolic syndrome group (MetS) and HFD for 2 weeks as the initial metabolic syndrome group (I-MetS). WT mice were obtained from Charles River Laboratories (Sulzfeld, Germany). During isoflurane anesthesia mice were fixed on a heat-controlled plate and an intra-arterial pressure transducer was inserted in the left carotid artery under sterile conditions. Intra-arterial blood pressure was measured continuously for 15 minutes (Blood Pressure Monitor BP1, World Precision Instruments, Sarasota, FL, USA). Thereafter, animals were sacrificed and the entire intestine, including vascular arcades, was immediately excised and stored in cold 3-(*N*-morpholino)propanesulfonic acid (MOPS) buffer for wire myograph studies. Changes in whole body weight and epididymal fat pad weight were assessed as parameters of visceral fat status. Serum was collected in serum separator tubes (Sarstedt, Nümbrecht, Germany) and stored at −80°C for subsequent experiments. All animals received humane care and all animal protocols were fully compliant with the principles of the Guide for the Care and Use of Laboratory Animals from the National Institutes of Health and German Law on the Protection of Animals was followed. The protocol was approved by the government of Bayern’s Animal Care Committee, Regierung von Oberbayern, Munich, Germany (Protocol number: AZ 55.2-1-54-2531-19-09). The mice were observed daily for any signs of distress and weighed weekly to monitor health. Blood pressure measurement in the carotid artery was performed under isoflurane anesthesia, and termination was performed with cervical dislocation. All efforts were made to minimize suffering.

### Magnetic resonance imaging

Whole body magnetic resonance imaging (MRI) was performed on mice anesthetized with intraperitoneal pentobarbital and placed in the prone position on a 47-mm microscopy surface coil inside the clinical 1.5 T MRI System (Achieva 1.5 T, Philips Medical Systems, Best, The Netherlands). An axial, multi-slice, turbo spin echo sequence [resolution 0.25 × 0.25 × 0.35 mm^3^, 140 slices, echo time (TE) = 100 ms, repetition time (TR) = 1000 ms was applied to suppress signal from tissue other than fat. The whole body images were reconstructed using an OsiriX DICOM viewer.

### Biochemical measurements

Serum fasting total cholesterol, triglycerides, and glucose levels were measured by enzymatic methods (Roche Diagnostics). Fasting insulin was measured by enzyme-linked immunosorbent assay (ELISA) kit (Shibayagi, Shibukawa, Japan). The insulin resistance index [homeostasis model assessment (HOMA-IR)] was calculated using fasting insulin and glucose values: [insulin (picomoles per liter) × glucose (millimoles per liter)]/22.5 [30,31]. Serum tumor necrosis factor-α (TNF-α) and interleukin-6 (IL-6) were measured with murine ELISA kits (Peprotech, Hamburg, Germany). Dissected mesenteric PVAT was snap-frozen and protein extracts were used for Rho-associated kinase activity measurement by an enzyme immunoassay (Cell Biolabs, San Diego, CA, USA). Measurements were performed according to the manufacturer’s protocol using 10 μg of protein lysate. Values are reported as percentage of activity related to controls.

### Histology

Epididymal fat pads and mesenteric vascular beds with PVAT were fixed in paraformaldehyde, embedded in paraffin, and subsequently stained with hematoxylin-eosin. For each mouse, the area of 50 randomly chosen adipocytes was measured in five representative sections using Image J software (National Institutes of Health, USA) at 10× magnification. Mean values given in pixels were compared. Analysis of infiltrating leukocytes in mesenteric PVAT was performed by immunohistochemical staining for CD45 (Becton & Dickenson, Franklin Lakes, NJ, USA). The total number of CD45-positive cells was counted in ten randomly chosen arteries per whole mesenteric vascular bed per mouse and the average calculated.

### Two-photon microscopy

Small mesenteric arteries and correspondent PVAT were visualized using a Leica SP5 II MP two-photon laser scanning microscope coupled with a water dipping 20× NA 1.00 objective and a pre-chirped Ti:Sa laser (Spectra Physics, Springfield, OH, USA) tuned to 840 nm [32]. Four hybrid detectors (HyD) were spectrally tuned for optimal detection efficiency and low bleed through of signal: second harmonic generation of collagen (HyD1: 400–425 nm), autofluorescence of adipocytes and GR1/eFluor450 (HyD2: 445–500 nm), autofluorescence of adipocytes/elastin, and CD115/Alexa488 (HyD3: 515–555 nm) CD45/nanocrystal-605 nm (HyD4: 590–625 nm). Additional image processing was performed using Leica LAF AF 3.0 and ImagePro. Quantification of inflammatory cells was performed by detecting the number of cells positive for CD45/CD115 (monocyte and macrophage marker) and for CD45/Gr-1 (marker for neutrophil in peripheral organs) in each arterial segment recorded in 3D datasets (*n* = 3 arterial segments per mouse, *n* = 4 mice per group). All antibodies were from eBioscience (San Diego, CA, USA). We counted the number of inflammatory cells using 3D datasets from the PVAT of the small mesenteric arteries (up to a distance of three times the average adipocyte diameter from the small artery). The adipocyte volume was determined by measuring maximal diameter in the corresponding arterial segment, assuming a spherical cell shape. The total number of positive cells is presented as inflammatory cells per adipocyte.

### RNA isolation and real-time polymerase chain reaction

Dissected mesenteric PVAT was snap-frozen in liquid nitrogen and stored at −80°C. Then, 80 mg of tissue was used for RNA isolation using RNeasy Lipid Tissue mini kit (Qiagen, Hilden, Germany) according to the manufacturer’s protocol. Quality of RNA was assessed using Bioanalyzer (BioRad, Hercules, CA, USA). Complementary DNA was synthesized using iScript cDNA Synthesis Kit (BioRad), according to the manufacturer’s protocol. Quantitative reverse transcriptase polymerase chain reaction (PCR) was performed using SYBR Green I (MyiQ ICycler, Bio-Rad). Gene expression of TNF-α, IL-6, monocyte chemotactic protein-1 (MCP-1), Toll-like receptor 4 (TLR4), RhoA, Rho-kinase 1 (ROCK1), and Rho-kinase 2 (ROCK2) were investigated. cDNA primers (sense and anti-sense) were as follows: glyceraldehyde 3-phosphate dehydrogenase (GAPDH) (TCGGTGTGAACGGATTTGGC and TTTGGCTCCACCCTTCAAGTG), TNF-α (CCAAAGGGATGAGAAGTTCC and GGCAGAGAGGAGGTTGACTTT), IL-6 (CTGGGAAATCGTGGAAATGAG and ACTCTGGCTTTGTCTTTCTTG), MCP-1 (GCTGTAGTTTTTGTCACCAAG and GATTTACGGGTCAACTTCACA), TLR4 (ATTCCCTCAGCACTCTTGATT and AGTTGCCGTTTCTTGTTCTTC), RhoA (CTCTCTTATCCAGACACCGAT and CAAAAACCTCTCTCACTCCATC), ROCK 1 (AAGGCGGTGATGGCTATTATG and TCCTCTACACCATTTCTGCCC), and ROCK 2 (ATGTGATTGGTGGTCTGTAGGT and AGCTGCCGTCTCTCTTATGTTA). Quantification was made using the ddCt algorithm, including normalization of each sample to GAPDH [33]. The results are expressed as the multiple of the control value from three independent experiments.

### Western blot

Total adiponectin and adiponectin multimers were determined by Western blotting in serum. Sodium dodecyl sulfate polyacrylamide gel electrophoresis (SDS**-**PAGE) was performed. In brief, serum proteins were separated by 10% SDS-PAGE under non-reducing and non-heating conditions, and transferred to nitrocellulose membranes. Membranes were blocked with Tris-buffered saline-Tween 20 containing 5% skim milk and incubated with a goat anti-human adiponectin polyclonal antibody (1:500). After washing, membranes were incubated with horseradish peroxidase conjugated-donkey anti-goat antibody (1:4000). Bands were visualized by using lumi-light Western blotting substrate, and the image was acquired with a Kodak IS440CF Imaging Station. Densitometry analysis was performed with Adobe Photoshop software. Relative distributions of adiponectin multimers were calculated by dividing band density by total density.

### Wire myograph and microvascular studies

First- to second-order branches from the superior mesenteric artery (270–330 μm) were cleaned of PVAT, cut into 2-mm-long rings, and mounted in a 4-channel wire myograph (Model 620 M, Danish Myo Technology, Aarhus, Denmark). Each vessel segment was mounted on two tungsten wires (40 μm diameter) in the organ chamber filled with MOPS buffer. MOPS buffer consisted of (in mM): NaCl 145, KCl 4.7, CaCl_2_ 3.0, MgSO_4_ 1.17, NaH_2_PO_4_ 1.2, pyruvate 2.0, EDTA 0.02, MOPS 3.0, and glucose 5.0. Vessels were pre-stretched to a tension representing a blood pressure of 13.3 kilopascal and equilibrated at this tension for 30 minutes at 37°C [34]. Subsequently, the organ bath solution was changed for a fresh pre-heated MOPS buffer and vascular functions were analyzed. During the experiments, the diameter of the vessels was kept constant, so the vessels could be examined under isometric conditions. For testing viability, vessels were subjected to norepinephrine-induced constriction followed by acetylcholine. Vessels with endothelium-dependent relaxation in the presence of acetylcholine that was greater than 70% of the maximal norepinephrine vasoconstriction were considered to have an intact endothelium. After washing out with MOPS buffer and resting for 20 min, norepinephrine (10^−9^ to 10^−5^ M) and acetylcholine (10^−10^ to 10^−5^ M) dose–response curves were constructed. Relaxation of preconstricted (high potassium chloride, 125 mM) vessels in response to an external NO donor (sodium nitroprusside, 10^−10^ to 10^−5^ M) was measured. Response was expressed as a percentage of potassium-induced constriction.

In the set of experiments designed to study the influence of PVAT on vascular response, one segment of a small mesenteric artery was cleaned of PVAT while the adjacent segment was left uncleaned. Arteries were first constricted with norepinephrine (10^−5^ M) to obtain the baseline response. After washing and resting for 20 min, norepinephrine dose–response curves were constructed, where the response was expressed as a percentage of the baseline norepinephrine-induced contraction.

### Statistical analysis

All data are presented as mean ± standard error of the mean (SEM). In cumulative dose–response curves of myograph experiments, the logEC50 value for tissue from each mouse (two to four arteries per mouse) was calculated. Differences between logEC50 values were calculated using non-linear regression analysis. In other experiments, comparisons between two groups were made with Student’s *t*-test. For comparisons between more than two groups, one-way ANOVA with Tukey post-hoc test was used. A probability value of <0.05 was considered statistically significant (GraphPad Prism ® 5.0).

## Results

### A mouse model to investigate the initial phase of metabolic syndrome

**Figure 1:**
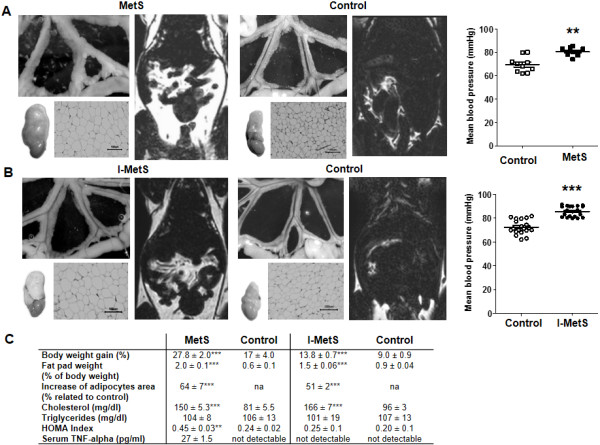
**Features of metabolic syndrome in WT mice. A**, After a HFD for 8 weeks (Metabolic Syndrome = MetS) and **B**, 2 weeks (Initial Metabolic Syndrome = I-MetS), there was increase in visceral fat tissue seen in mesenteric PVAT, epididymal fat pads, adipocyte hypertrophy and hyperintense areas displaying fat depots in magnetic resonance imaging. Elevation of blood pressure was seen in MetS and I-MetS groups. **C**, Comparative table of weight and metabolic parameters (n = 10–15 mice/group). Data are presented as mean ± SEM, n = 10-20 mice/group, **P <0.01, ***P < 0.001, na = not applicable.

A high-fat diet was used to induce features of MetS in WT mice. As expected, WT mice fed a HFD for 8 weeks (MetS group) gained a significant amount of weight, and showed a marked increase in visceral fat tissue (mesenteric PVAT and epididymal fat pads) and hypertrophy of adipocytes. Mean arterial blood pressure was significantly elevated (control: 70.5 ± 2.1 mmHg vs. MetS: 82.7 ± 1.6 mmHg, *P* < 0.01) (Figure 1A). MetS group mice also presented with insulin resistance and systemic inflammation, as measured by increases in HOMA-IR and serum TNF-α level, respectively (Figure 1C).

Compared with controls, mice fed a HFD for 2 weeks (I-MetS group) also had significantly heavier epididymal fat pads and increased mesenteric PVAT, accompanied by adipocyte hypertrophy (Figure 1B). However, disorders associated with MetS, such as insulin resistance and systemic inflammation, were not present (Figure 1C). Mean arterial blood pressure was significantly elevated in I-MetS group mice, as was observed in the MetS group (control: 72 ± 1.3 mmHg vs. I-MetS: 85 ± 1.0 mmHg, *P* < 0.001) (Figure 1B).

### The initial phase of MetS presents with augmented vasoconstriction, independent of endothelium and PVAT

**Figure 2:**
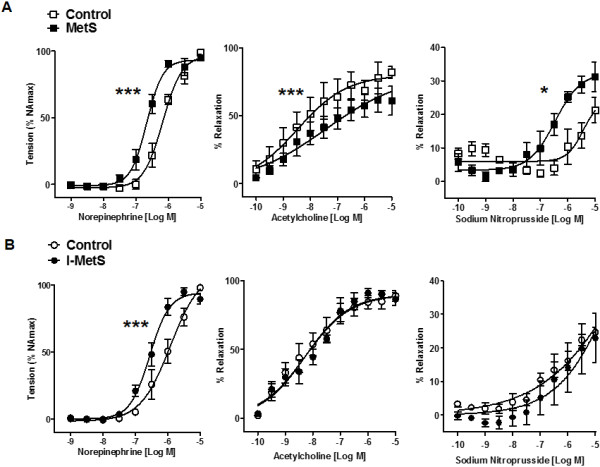
**Microvascular function in MetS and I-MetS mice. A**, Norepinephrine-induced vasoconstriction was significantly increased, acetylcholine-induced relaxation was reduced and responses to sodium nitroprusside were increased in MetS mice. **B**, Similarly to MetS, the I-MetS group also presented increased vasoconstriction but without differences in relaxation responses. Data are presented as mean ± SEM, n = 10 mice/group, *P < 0.05, ***P < 0.001.

**Figure 3:**
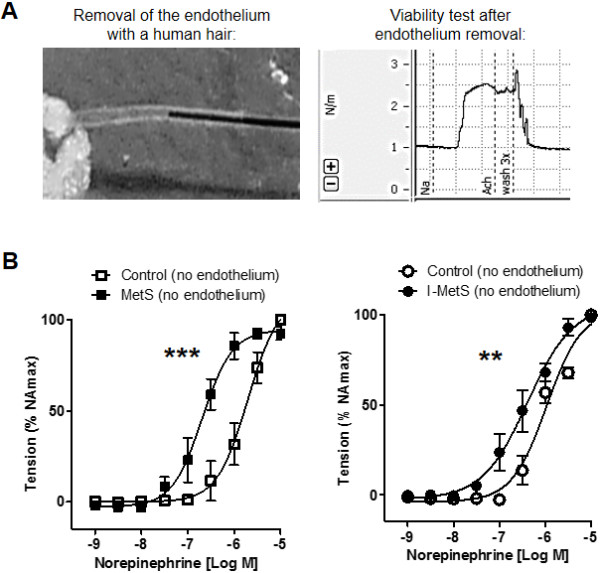
**Effect of endothelium removal on vasoconstriction responses. A**, Mechanical removal of endothelium with a human hair and viability test showing no relaxation response to acetylcholine. **B**, Endothelium-denuded arteries from both MetS and I-MetS groups still presented significantly increased vasoconstriction responses compared to controls. Data are presented as mean ± SEM, n = 4–5 mice/group, **P <0.01, ***P < 0.001.

In MetS mice, small mesenteric arteries had an augmented response to the vasoconstrictor norepinephrine, demonstrated by measurement of sensitivity (control: logEC50 −6.170 vs. MetS: logEC50 −6.664, *P* < 0.001). In MetS mice, relaxation in response to acetylcholine was significantly lower compared with control, and that to sodium nitroprusside was significantly augmented (Figure 2A). I-MetS group mice also had increased sensitivity to norepinephrine (control: logEC50 −5.921 vs. I-MetS: logEC50 −6.556, *P* < 0.001), but relaxation responses to acetylcholine and sodium nitroprusside were the same as seen in control mice (Figure 2B). Mechanical removal of the endothelium (Figure 3A) did not affect the pattern of vasoconstriction responses in arteries from MetS and I-MetS mice, suggesting that these vascular findings are at least partially independent of endothelium integrity (Figure 3B).

**Figure 4:**
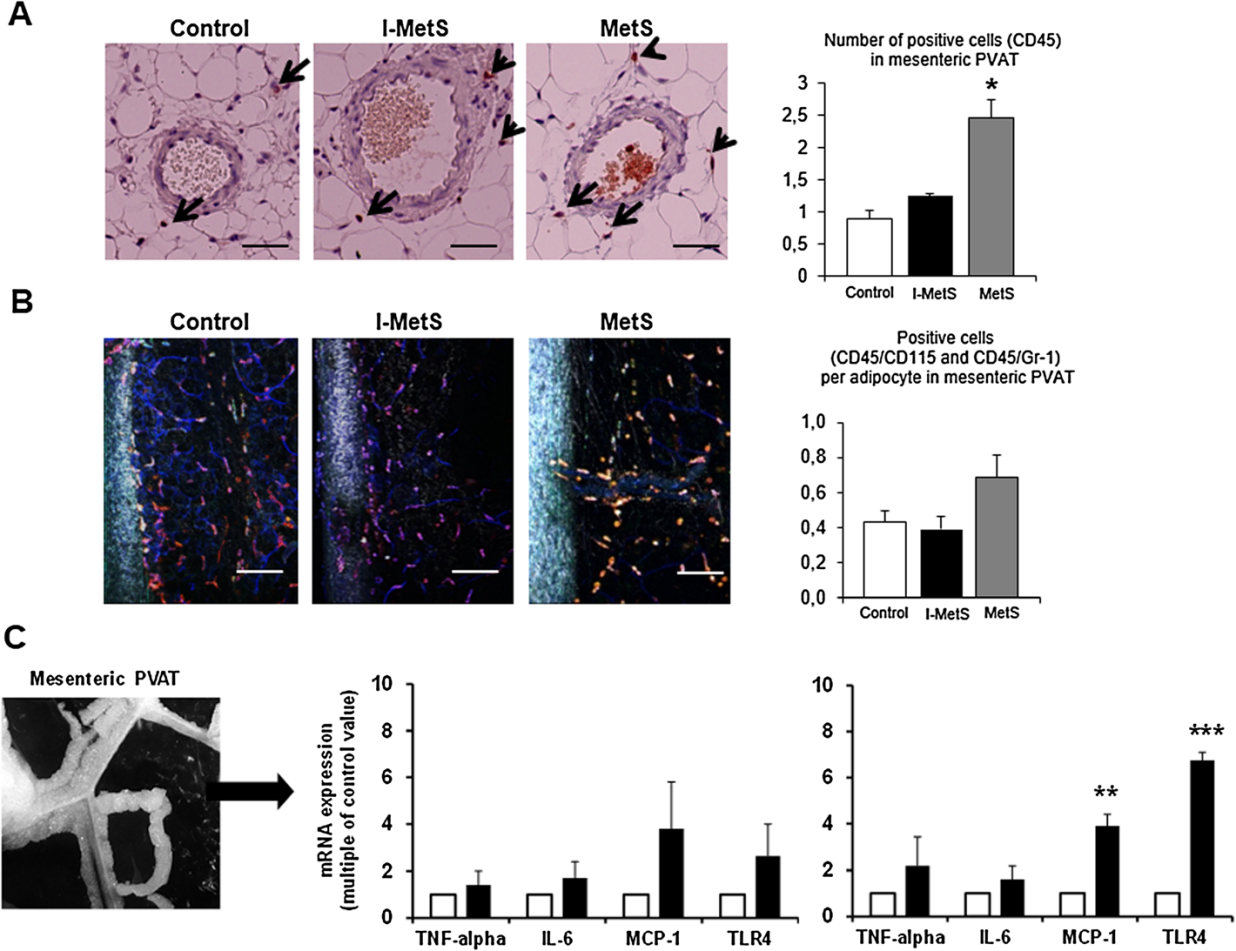
**Inflammatory cell infiltration in mesenteric perivascular adipose tissue (PVAT). A**, Representative images of CD45 immunohistochemical staining of a small mesenteric artery and surrounding PVAT. The number of CD45-positive cells was not different between I-MetS mice and controls, but it was increased in MetS mice (data are presented as mean ± SEM, n = 6 mice/group, *P < 0.05, scale bars = 50 μm). **B**, Representative images of two-photon microscopy of a small mesenteric artery on the left, with the surrounding PVAT stained for CD45/CD115 and CD45/Gr-1. I-MetS mice did not present with increased number of positive cells. In MetS mice there was a trend toward more positive cells (*P* = 0.06, scale bars = 100 μm). The column graph represents the number of positive cells per arterial segment examined (data are presented as mean ± SEM, 3 segments/mouse, n = 4 mice/group). **C**, In contrast with MetS mice, I-MetS mice did not present increased expression of inflammatory genes in mesenteric PVAT. Results represent three independent experiments, n = 3–4 mice/group, data are presented as mean ± SEM, *P < 0.05, **P < 0.01.

Next, we investigated mesenteric PVAT. Infiltration by inflammatory cells was first investigated by immunohistochemistry. The number of cells positive for the pan-leukocyte marker CD45 was increased only in MetS mice and not in the I-MetS group (Figure 4A). Two-photon microscopic imaging of small mesenteric arteries surrounded by PVAT showed that the number of cells positive for CD45/CD115 (monocytes and macrophages) and CD45/Gr-1 (neutrophils) tended to increase (*P* = 0.06) in the PVAT from just MetS mice (Figure 4B). Gene expression of inflammatory markers, such as MCP-1 and TLR4, were significantly increased in the PVAT from just MetS mice (Figure 4C).

**Figure 5:**
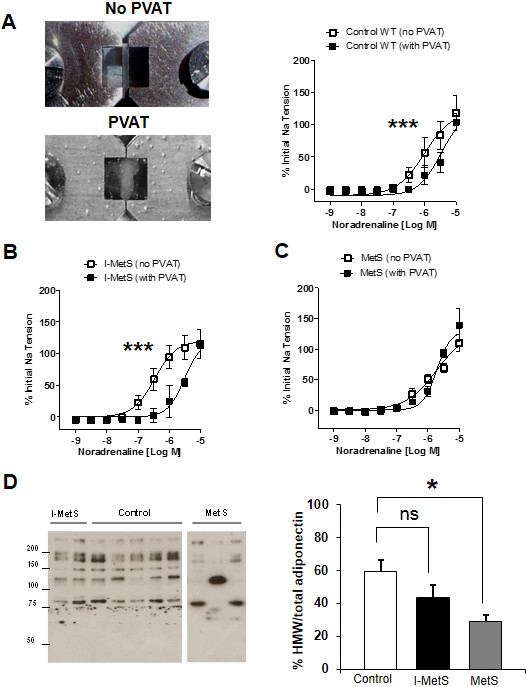
**Role of PVAT in vascular responses and adiponectin measurement. A**, Arteries examined in the myograph without and with PVAT, control mice. **B**, I-MetS mice showed PVAT with anticontractile properties similar to control mice. **C**, MetS mice did not present with this effect anymore (Data presented as mean ± SEM, n = 4 mice/group, ***P < 0.001). **D**, Lower levels of high-molecular weight (HMW) adiponectin in relation to total adiponectin in serum were seen only in MetS mice (Data presented as mean ± SEM, n = 5 mice/group, *P <0.05, ns = not significant).

**Figure 6:**
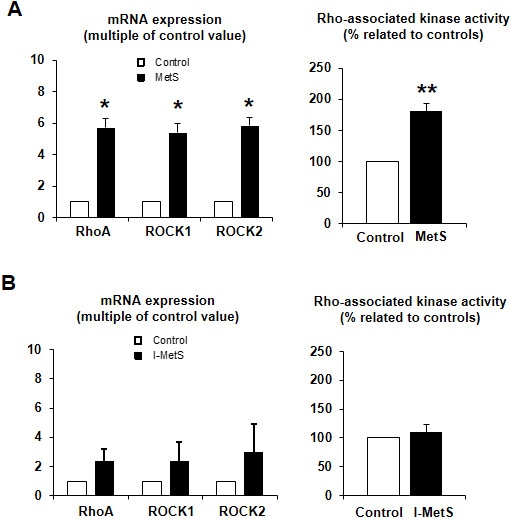
**Activation of RhoA/Rho-kinase pathway in mesenteric PVAT. A**, Mesenteric PVAT from MetS mice showed increased gene expression of RhoA and Rho-kinases. Rho-kinase activity was also significantly increased, which was not seen in I-MetS mice **(B)**. Data represent the mean ± SEM from 2–4 independent experiments, n = 3–4 mice/group, *P < 0.05, **P < 0.01.

To investigate the influence of PVAT in vasoconstriction responses we examined arteries with and without this tissue. In control mice, PVAT has an anticontractile effect (Figure 5A). The data obtained strongly suggest that, in I-MetS mice, PVAT keeps its anticontractile properties (Figure 5B), whereas the MetS group has lost this characteristic (Figure 5C). Among the reported PVAT-derived effectors that can influence vascular contractility are adiponectin levels and activation of the RhoA/Rho-kinase pathway. Adiponectin is present in the circulation as three distinct oligomeric complexes [35] and it has been described as having a key role in modulating vascular function [16]. However, in I-MetS mice, levels of adiponectin, and in particular of high-molecular weight (HMW) adiponectin, were unchanged compared with controls. In contrast, MetS mice had significantly reduced HMW adiponectin (Figure 5D). In the PVAT from MetS mice (Figure 6A), gene expression of the small GTPase RhoA and of two of its downstream effectors, ROCK1 and ROCK2, was increased. In MetS mice Rho-kinase activity was increased, but this was not observed in the PVAT from I-MetS mice (Figure 6B). To summarize, in contrast with MetS mice, I-MetS mice exhibited augmented vasoconstriction that was independent of PVAT and some of its described vasoactive effectors.

## Discussion

This study was designed to investigate microvascular function during the development of MetS induced by HFD. We showed that microvascular dysfunction in the form of augmented vasoconstriction was already present in the initial phase of MetS, as was blood pressure elevation. These early vascular alterations developed in the absence of important factors known to influence microvascular function, such as insulin resistance, systemic inflammation, and endothelial and PVAT dysfunction, which were only observed in a later phase of MetS.

### Microvascular dysfunction was present already in the initial phase of metabolic syndrome

Prolonged HFD in C57BL/6 mice is an established model of diet-induced MetS [26]. In our study I-MetS mice presented with mild weight gain, a prominent visceral fat increase, hypercholesterolemia, and blood pressure elevation; however, systemic inflammation and insulin resistance were not observed. After 8 weeks of HFD, mice presented with features similar to those found in human MetS, such as weight gain with a marked increase in visceral fat, hypercholesterolemia, and blood pressure elevation. In addition to these classical components, MetS mice presented with insulin resistance and systemic inflammation indicated by elevated TNF-α and reduced adiponectin levels. These factors are part of the additional metabolic criteria suggested for research by the International Diabetes Federation, aiming at further modification of the MetS definition [36].

Concerning the microvasculature, MetS mice presented with augmented vasoconstriction responses to norepinephrine, while the relaxation responses to acetylcholine and sodium nitroprusside were reduced and augmented, respectively, suggesting endothelial dysfunction. Similarly, augmented vasoconstriction in the presence of norepinephrine was present in I-MetS mice. However, contrary to MetS mice, relaxation induced by acetylcholine and sodium nitroprusside was preserved in I-MetS mice, suggesting the integrity of endothelial function. Obesity is reported to be associated with impaired NO bioavailability with a compensatory increase in the activity of soluble guanylyl cyclase [37], the major cellular receptor for NO. The augmented relaxation responses to sodium nitroprusside, an external source of NO, suggest impaired NO bioavailability and, together with decreased relaxation responses to acetylcholine, strongly suggest diminished endothelial function. Therefore, augmented vasoconstriction in the absence of endothelial dysfunction as observed in I-MetS mice strengthens the evidence of a role for vascular smooth muscle cells. It is known that endothelial function can be influenced by the presence of insulin resistance [14], systemic inflammation [38], and dysfunctional adipose tissue [16,39]; therefore, association of these factors in MetS mice was expected. In contrast to these findings in MetS mice, the augmented vasoconstriction in I-MetS mice was independent of endothelial dysfunction, insulin resistance, and systemic inflammation.

Human MetS has been associated with an increased risk of CVD such as myocardial infarction, stroke [40], and coronary microvascular dysfunction linked to a microvascular form of angina pectoris [11]. Microvascular dysfunction is reported to be associated with poor cardiovascular outcomes [41,42], including left ventricular remodeling [43]. Thus, identification of specific MetS components that are associated with microvascular dysfunction might help illuminate the pathophysiology of CVD in MetS. The community-based Bogalusa Heart Study evaluated normotensive, prehypertensive, and hypertensive adults. The study showed that the early natural history of hypertension was characterized by excess adiposity and blood pressure beginning in childhood and unfavourable changes in risk variables of MetS occuring through young adulthood. Importantly, our study showed that, as in MetS mice, I-MetS mice presented with blood pressure elevation plus augmented vasoconstriction, suggesting that there is a close relationship between these features because the microcirculation actively regulates blood pressure control [8]. Therefore, augmented vasoconstriction could underlie the onset of hypertension in MetS.

### Dysfunction of adipose tissue was absent in the initial phase of metabolic syndrome

The vasoactive properties of PVAT are now recognized as being protective in healthy conditions [16]. However, in the setting of obesity, for example, inflammation in adipose tissue, such as that caused by macrophage activation [44], contributes to the loss of this protective vascular effect [23,45]. In our study, mice with I-MetS did not show signs of dysfunctional PVAT, presenting with neither infiltration by inflammatory cells nor increased expression of inflammatory genes. Loss of the anticontractile effect was only seen in MetS mice and not in the I-MetS group. Activation of the renin-angiotensin–aldosterone system [46,47] is involved in diet-induced obesity, since hypertrophied adipocytes can serve as a source of angiotensin II [48]. Although our experiments do not rule out the role of angiotensin II in early stages of disease development, the PVAT had a protective role in vasoconstriction responses in I-MetS mice. This observation is at odds with the potential deleterious effect of angiotensin II.

Adiponectin has been addressed as an important vasodilator effector secreted from PVAT, and Greenstein et al. reported loss of the protective vasodilator effect of PVAT involving adiponectin in obese patients [16]. Moreover, lower levels of adiponectin, in particular HMW adiponectin, have been linked to MetS [49]. We observed decreased HMW adiponectin levels only in serum of MetS mice.

Another aspect related to PVAT addressed in this study was activation of the RhoA/Rho-kinase pathway, a key modulator of vascular smooth muscle contraction [50] and contributor to the pathophysiology of arterial hypertension [51]. Rho-kinase activity was reported to be upregulated in leukocytes from patients with MetS [52] and hypertension [53]. In animal models of hypertension, increased Rho-kinase activity was detected in vascular tissues [54,55]. More recently Hara et al. showed that stretching of adipocytes by accumulated lipid triggers RhoA/Rho-kinase signaling and the subsequent expression of inflammatory genes. Therefore, RhoA/Rho-kinase signaling is implicated in inflammatory changes in adipose tissue in obesity, contributing to and aggravating weight gain and insulin resistance [56]. Thus, it is plausible to hypothesize that signaling originating from PVAT influences vascular contractility. Interestingly, we saw that although PVAT from I-MetS mice was already hypertrophied there was no evidence of Rho-kinase activation; the latter was seen only in MetS mice, suggesting that the augmented vasoconstriction in I-MetS occurred independently of PVAT-derived RhoA/Rho-kinase signaling.

## Conclusion

In summary, this study identified distinct patterns of microvascular dysfunction in MetS, with augmented vasoconstriction present in the initial phase of MetS independent of endothelial dysfunction, insulin resistance, inflammation, and a dysfunctional PVAT, features commonly accepted as modulators of microvascular function. Our findings strongly suggest that, in I-MetS, the augmented vasoconstriction was caused by vascular smooth muscle cell dysfunction. These results shed light on the link between MetS and increased CVD risk. We cannot state the relevance of these results to human MetS. Further research is needed to identify the mechanisms of early microvascular changes and to provide improved preventive strategies aiming to decrease the CVD risk associated with MetS.

## Abbreviations

CVD: Cardiovascular diseases; EDTA: Ethylenediaminetetraacetic acid; ELISA: Enzyme-linked immunosorbent assay; GAPDH: Glyceraldehyde 3-phosphate dehydrogenase; HFD: High-fat diet; HMW: High molecular weight; HOMA-IR: Homeostasis model assessment-insulin resistance index; HyD: Hybrid detectors; IL-6: Interleukin-6; I-MetS: Initial metabolic syndrome; MCP-1: Monocyte chemotactic protein-1; MetS: Metabolic syndrome; MOPS: 3-(*N*-morpholino)propanesulfonic acid; MRI: Magnetic resonance imaging; NO: Nitric oxide; PCR: Polymerase chain reaction; PVAT: Perivascular adipose tissue; ROCK1: Rho-kinase 1; ROCK2: Rho-kinase 2; SDS-PAGE: Sodium dodecyl sulfate polyacrylamide gel electrophoresis; TE: Echo time; TLR4: Toll-like receptor 4; TNF-α: Tumor necrosis factor-α; WT: Wild type.

## Competing interests

The authors declare that they have no competing interests.

## Authors’ contributions

CA generated data, analyzed the data and wrote the manuscript. CA and MB designed the research and wrote the manuscript. RM, IH and RB generated data. SC, ES, RE, DS, CS, CW, UH, MT contributed to discussions and reviewed the manuscript. CA and MB are the guarantors of this work and, as such, had full access to all the data in the study and takes responsibility for the integrity of the data and the accuracy of the data analysis. All authors read and approved the final manuscript.
